# The Role of Nicotinamide as Chemo-Preventive Agent in NMSCs: A Systematic Review and Meta-Analysis

**DOI:** 10.3390/nu16010100

**Published:** 2023-12-27

**Authors:** Giulio Tosti, Francesca Pepe, Patrizia Gnagnarella, Flavia Silvestri, Aurora Gaeta, Paola Queirolo, Sara Gandini

**Affiliations:** 1 Dermato-Oncology Unit, European Institute of Oncology IRCCS, 20141 Milan, Italy; giulio.tosti@ieo.it (G.T.); francesca.pepe@ieo.it (F.P.); flavia.silvestri@ieo.it (F.S.); 2 Division of Epidemiology and Biostatistics, European Institute of Oncology IRCCS, 20141 Milan, Italy; 3 Molecular and Pharmaco-Epidemiology Unit, Department of Experimental Oncology, European Institute of Oncology IRCCS, 20139 Milan, Italy; aurora.gaeta@ieo.it (A.G.); sara.gandini@ieo.it (S.G.); 4 Department of Statistics and Quantitative Methods, University of Milan-Bicocca 8, 20126 Milan, Italy; 5 Division of Medical Oncology for Melanoma, Sarcoma, and Rare Tumors, European Institute of Oncology IRCCS, 20141 Milan, Italy; paola.queirolo@ieo.it

**Keywords:** non-melanoma skin cancers, actinic keratoses, basal cell carcinoma, squamous cell carcinoma, nicotinamide

## Abstract

Background: Nicotinamide is the active form of vitamin B3 (niacin) obtained through endogenous synthesis, mainly through tryptophan metabolism and dietary supplements, fish, meats, grains, and dairy products. It participates in cellular energy metabolism and modulates multiple cellular survival and death pathways. Nicotinamide has been widely studied as a safe chemopreventive agent that reduces actinic keratosis (AKs) and non-melanoma skin cancers (NMSC). Methods: We used the Medline, EMBASE, PubMed, and Cochrane databases to search the concepts “nicotinamide”, “chemoprevention”, and “skin cancer” up to August 2023. Three independent authors screened titles and abstracts for intervention and study design before searching full texts for eligibility criteria. The primary outcome was the impact of oral nicotinamide on the incidence of NMSC in high-risk patients. We also conducted a systematic search to identify relevant epidemiological studies published evaluating dietary niacin intake and the risk of NMSC. Results: Two hundred and twenty-five studies were reviewed, and four met the inclusion criteria. There was no association between NAM consumption and risk for squamous cell carcinoma (SCC) (rate ratio (RR) 0.81, 95% CI 0.48–1.37; I^2^ = 0%), basal cell carcinoma (BCC) (RR 0.88, 95% CI 0.50–1.55; I^2^ = 63%), and NMSC (RR 0.82, 95% CI 0.61–1.12; I^2^ = 63%). Adverse events were rare and acceptable, allowing optimal compliance of patients to the treatment. We found only one article evaluating the association between niacin dietary intake and NMSC risk, supporting a potential beneficial role of niacin intake concerning SCC but not BCC or melanoma. Conclusions: The present meta-analysis shows, by pooling immunocompetent and immunosuppressed patients, that there is insufficient evidence that oral nicotinamide therapy significantly reduces the number of keratinocyte cancers.

## 1. Introduction

Non-melanoma skin cancer (NMSC) is the most prevalent type of cancer, representing about one-third of all malignancies diagnosed worldwide yearly [1]. NMSC is the most common cancer in fair-skinned people, including basal cell carcinoma (BCC) and squamous cell carcinoma (SCC) [2]. The age-standardized incidence rate of NMSC increased from 54.08/100,000 (95% uncertainty interval (UI): 46.97, 62.08) in 1990 to 79.10/100,000 (95% UI: 72.29, 86.63) in 2019, with an estimated percentage change of 1.78 per year [1].

Actinic keratoses (AKs) are keratinocyte neoplasms occurring on chronically sun-damaged skin that might spontaneously evolve into squamous cell carcinoma (SCC). Preventative treatment and photoprotection measures are recommended, given the elevated prevalence of AKs and the potential for progression [3].

In recent years, numerous studies have investigated the role of nicotinamide or niacinamide (NAM) in cancer chemoprevention. NAM is an amide form of vitamin B3, one of two primary forms of vitamin B3 (niacin or nicotinic acid). Niacin and NAM are substrates for two coenzymes: nicotinamide adenine dinucleotide (NAD) and NAD phosphate (NADP). NAD and NADP are electron acceptors for energy molecules in redox reactions. Intracellular NAD+ levels are modulated by nicotinamide N-methyltransferase (NNMT), a cytosolic enzyme critical for the destiny of NAM. NNMT catalyzes the methylation of NAM to 1-methyl nicotinamide (MNA) so that MNA cannot join the NAD+ salvage pathway, decreasing the intracellular NAD+ levels [4]. NNMT is broadly explored in cancer, and it was found to be upregulated in skin cancer. Niacin exerts antioxidant effects and converts nutrients to energy, DNA synthesis and repair, and several cellular activities [5]. For this reason, it has been hypothesized to shield cells from oxidative stress and to enhance cell survival [6,7,8].

A growing body of proof has revealed that maintenance of the cellular nicotinamide adenine dinucleotide phosphate (NADPH) content is implicated in a variety of pathological conditions, such as diabetes, cardiovascular diseases, neurodegenerative disorders, tumorigenesis, and cancer progression [9,10]. Compared with non-tumor cells, tumor cells usually keep elevated levels of NADPH to power redox protection and use biosynthetic reactions to sustain their fast expansion [11]. Among the metabolic paths regulating the cytosolic availability of NADPH, the pentose phosphate pathway plays a meaningful role, utilizing Glucose-6-phosphate dehydrogenase (G6PD) and 6-phosphogluconate dehydrogenase (PGD). NADPH production is dramatically increased by improving the flux of glucose into the pentose phosphate pathway oxidative branch in different cancer types [10]. G6PD deficiency significantly decreases NADPH levels and improves chemotherapeutic drug-induced apoptosis via redox modulation [10].

The association between niacin intake and chronic disease outcomes has been investigated in recent years. So far, it has not been feasible to uncover an association between niacin intake and mortality for any cause [12], cancers [13,14,15,16], cognitive function [17], bone health [5], and neurological conditions [18].

Nonetheless, evidence indicates that NAM has a role in the chemoprevention and therapy of some cancer types. For instance, the role of NAM in the chemoprevention of skin cancer has been widely studied, and recently, NAM was included among strategies for treating the field of cancerization [19].

Ultra-violet (UV) radiation is the primary etiologic agent in developing skin cancers, mainly NMSC, through direct cellular damage and skin immunity impairment [20]. NAM supports the energetic function of keratinocytes, being the precursor of NAD+ and increasing ATP production. This way, NAM enhances DNA repair and reduces UV-induced immunosuppression [21]. Moreover, at higher concentrations, NAM inhibits the nuclear DNA repair enzyme poly-ADP-ribose polymerase-1 (PARP-1), activated by UV radiation. When overexpressed, PARP-1 yields the loss of NAD, thus harming cellular metabolism and death [22,23].

We conducted a systematic review and meta-analysis to assess the dietary intake and role of NAM as a chemopreventive agent in NMSC, evaluating the efficacy and safety of the molecule.

## 2. Materials and Methods

The protocol of this systematic review is available online in the PROPSERO registry website (CRD42023443237, accessed on 1 August 2023).

A systematic literature search and quantitative analysis were planned, conducted, and reported following the Preferred Reporting Items for Systematic Reviews and Meta-analyses (PRISMA) [24] and CONSORT statements [25]. The inclusion criteria were based on the PICO’S framework [26].

### 2.1. Literature Search

Published reports were obtained from the following databases using validated search strategies: PMED (http://www.ncbi.nlm.nih.gov/entrez/query.fcgi accessed on 21 December 2023), Ovid Medline (Ovid Technologies, Inc., New York, NY, USA, 1950-29 April 2011), EMBASE (Elsevier, Amsterdam, The Netherlands, 1980-29 April 2011), and ISI Web of Knowledge (Thomson Scientific Technical Support, New York, NY, USA, 1945-4 May 2011 up to August 2023). We manually searched references cited in the retrieved articles and preceding reviews on the topic. Ecological studies, case reports, reviews, and editorials were not considered eligible. We screened titles and examined abstracts when the title suggested a study that conceivably met the three main criteria. If the abstract content was relevant, complete copies of articles were retrieved and fully read by at least two co-authors.

Three authors selected the articles (SG, PG, and FP).

The publications were rescued using the following two search strings:

((((prevent [tiab] OR prevents OR prevented OR preventing OR prevention[tiab] OR prophylax* OR prophylac* OR chemoprevention OR chemoprevent* OR thwart* OR “ward off” OR “ward-off” OR pre-emptive* OR preemptive* OR chemoprophyla* OR “prevention and control”[sh]) OR ((reduc* OR diminish* OR decreas* OR minimiz* OR minimis*) AND (occur* OR inciden* or rate* OR odds OR likelihood OR develop* OR frequenc*))) AND (niacinamide OR nicotinamide OR “nicotinic acid amide” OR “Nicotinic amide” OR “Vitamin B3” OR “Vitamin B-3” OR “Pyridinecarboxamide” OR “papulex” OR “Nicotinsäureamid” OR enduramide OR nicobion OR “vitamin PP”)) AND ((“Skin Neo-plasms/prevention and control”[Mesh]) OR (skin neoplasms OR cutaneous neoplasms OR skin cancer OR cutaneous cancer OR skin carcinoma OR “basal cell carcinoma*” OR “Carcinoma, Basal Cell”[Mesh] OR “Carcinoma, Squamous Cell”[Mesh] OR “squamous cell carcinoma*” OR cutaneous carcinoma OR cscc OR csccs OR scc OR sccs OR bcc OR bccs OR nmsc OR nmscs OR acanthoma))) AND (((randomized controlled trial[pt]) OR (controlled clinical trial[pt]) OR (randomi*[tiab]) OR (placebo[tiab]) OR (drug therapy[sh]) OR (randomly[tiab]) OR (trial[tiab]) OR (groups[tiab])) NOT (animals[mh] NOT humans[mh]));

And the following:

(food OR foods OR eat OR eats OR eating OR ate OR consumption[tiab] OR consume OR consumes OR consumed OR consuming OR diet OR diets OR dietary OR dieting OR dieted OR dietician* OR dietetic* OR nutrition OR nutritional OR nutrient* OR nutritious OR nutritionist*) AND ((occur* OR inciden* or rate* OR odds OR likelihood OR develop* OR frequenc*) AND (niacinamide OR nicotinamide OR “nicotinic acid amide” OR “Nicotinic amide” OR “Vitamin B3” OR “Vitamin B-3” OR “Pyridinecarboxamide” OR “papulex” OR “Nicotinsaureamid” OR enduramide OR nicobion OR “vitamin PP”) AND ((“Skin Neo-plasms/prevention and control”[Mesh]) OR (skin neoplasms OR cutaneous neoplasms OR skin cancer OR cutaneous cancer OR skin carcinoma OR “basal cell carcinoma*” OR “Carcinoma, Basal Cell”[Mesh] OR “Carcinoma, Squamous Cell”[Mesh] OR “squamous cell carcinoma*” OR cutaneous carcinoma OR cscc OR csccs OR scc OR sccs OR bcc OR bccs OR nmsc OR nmscs OR acanthoma))).

The database search was supplemented using forward citation chaining, i.e., by perusing the reference list of eligible papers and previously published reviews and meta-analyses.

We selected studies reporting the minimum information necessary to perform adequate meta-analysis:

1. Sufficient information to estimate the rate ratio (RR) and 95% confidence intervals (95%CI) of NAM vs. placebo for BCC and SCC.
2. Studies had to be independent and not duplicate results published in another article.

A standardized data-collection protocol was used to gather relevant data from each selected article.

There was no language, time, or geographical restriction.

Review articles not reporting original data were also excluded but were checked for references.

For each study selected for this review and meta-analysis, we retrieved information on the year of publication, country, study design, features of the enrolled sample, number of patients and events, treatment duration and compliance, route, dose, therapeutic scheme, and follow-up. When relevant information was unavailable in the manuscripts, we contacted the authors to retrieve it.

### 2.2. Statistical Analysis

The summary RR (SRR) was estimated by pooling the study-specific estimates with the random effects models described by van Houwelingen et al. [27], i.e., with summary effect size obtained from maximum likelihood estimation. Confidence intervals were computed consuming an underlying t-distribution. Between-study heterogeneity I^2^ was calculated and is presented with summary estimates [28]. A threshold of I^2^ below 50% is generally considered an acceptable level of variability.

Publication bias was evaluated graphically with a funnel plot, and we conducted the Macaskill test [29], which is more powerful when fewer than 20 estimates are included in the analysis.

The methodological quality of trials included in the meta-analysis was evaluated and rated using the Quality of Reporting of Meta-analyses (QUORUM) checklists [25] and the Cochrane risk-of-bias tool for clinical trials [30].

All the statistical analyses were performed using SAS software (SAS Institute Inc., Cary, NC, USA; version 9.2) and R software, version 2.12.2 (http://www.r-project.org accessed on 21 December 2023).

## 3. Results

### 3.1. Study Selection

**Figure 1:**
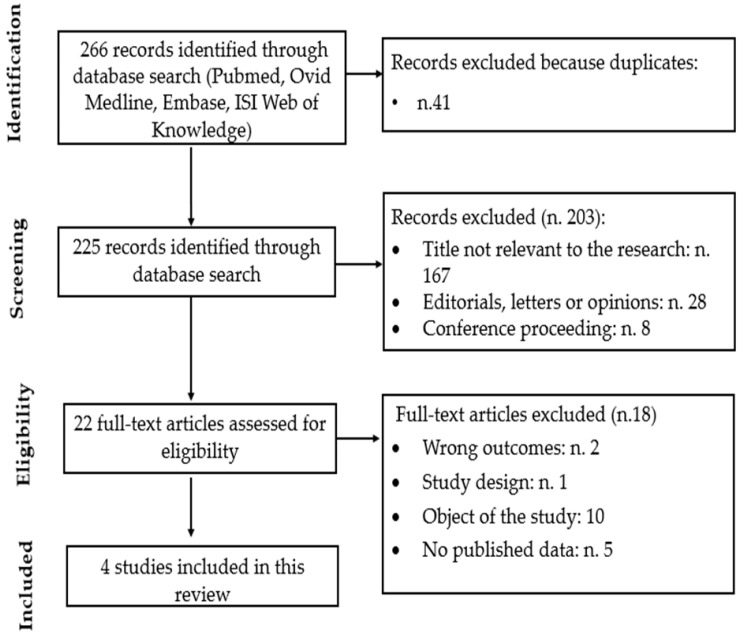
Study flow-chart for the process of selecting the enrolled studies.

The literature search in databases returned 266 items, which dropped to 225 after duplicates were removed (Figure 1). A total of 203 articles were removed based on their titles and abstracts, which left 22 papers to be read in full. Of these, 18 were removed for not matching the inclusion criteria. Thus, four independent studies were eligible. They were included in the review to report the association between the impact of NAM and the incidence of NMSC in high-risk patients.

### 3.2. Data Extraction

The main results and features of the selected studies are presented in Table 1. All the enclosed studies were double-blind, randomized trials carried out in Australia [31,32,33,34]. Two studies each were Phase II [31,34] and Phase III [32,33]. Three studies reported oral NAM posology of 500 mg twice daily; only in Surjana (2012) did randomized participants receive 500 mg of NAM once or twice daily (data presented in Table 1 are combined). Treatment duration varied from four [31] to twelve months [32,33]. Participants had at least two histologically confirmed NMSCs in the previous five years. Enrolled patients were immunocompetent in the studies by Chen (2015) and Surjana (2012), while Chen (2016) and Allen (2023) accrued immunosuppressed subjects, i.e., kidney-, liver-, heart-, or lung-transplanted subjects at least twelve months earlier. Treatment adherence was high in all studies; the lowest rate of adherence, 78%, was reported by Allen (2023).

### 3.3. Risk of Bias of Trials in Included in the Meta-Analysis

All studies clearly reported details (Surjana, 2012; Chen 2016; and Allen, 2023). PICO framework, scores of Cochrane risk-of-bias tool for clinical trials, and evidence-based GRADE level, evaluating the overall quality of the included studies, are presented in Table 2 [35,36].

3 **PICO**

- **Patient/Population/Problem**
  High-risk NMSCs patients
- **Intervention**
- Nicotinamide as chemopreventive agent
- **Comparison**
  Placebo/control
- **Outcome**(**s**)
  Incidence of new actinic keratosis/NMSC

### 3.4. Primary Outcome

**Figure 2:**
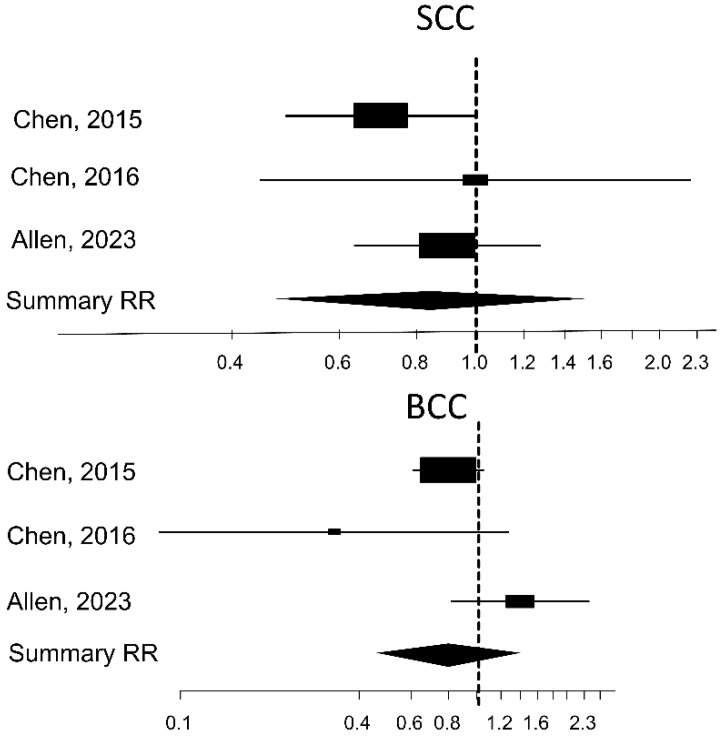
Forest plot of the measures of association between oral NAM and the risk of SCC and BCC (Chen, 2015 [32], 2016 [34]; Allen 2023 [33]).

**Figure 3:**
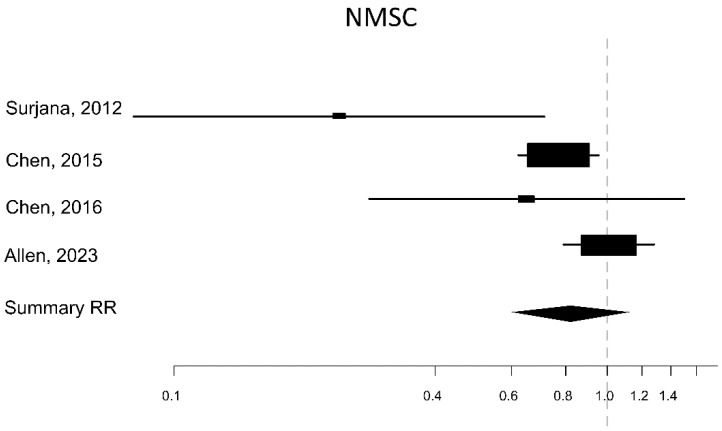
Forest plot of the measure of the association between oral NAM and the risk of NMSC (Surjana, 2012 [31]; Chen, 2015 [32]; Chen, 2016 [34]; Allen, 2023 [33]).

The meta-analysis revealed that there was no association between NAM consumption and risk for SCC (RR 0.81, 95% CI 0.48–1.37; I^2^ = 0%), BCC (RR 0.88, 95% CI 0.50–1.55; I^2^ = 63%) (Figure 2), and NMSC (RR 0.82, 95% CI 0.61–1.12; I^2^ = 63%) (Figure 3).

The high between-study heterogeneity (I^2^ > 50%) is mainly due to the significant protective effect found in the two first published clinical trials, Surjana 2012 and Chen 2015.

### 3.5. Other Studied Assessing the Effect of Nicotinamide with Actinic Keratoses (AK)

From five studies, it was impossible to extract the rate ratio estimate for NAM vs. placebo (Table 3), but they still presented some data on efficacy.

In two Phase II double-blind, randomized, controlled trials, preventive therapy with oral NAM reduced the onset of new AK lesions. NAM at the dose of 500 mg twice daily reduced AK count by 35% (*p* = 0.0006) after four months of treatment; NAM at the dose of 500 mg once a day lowered AK count by 29% (*p* = 0.005) [31].

In 2021, Ferreira et al. conducted an open, randomized, comparative, factorial, self-controlled, double-blind clinical trial investigating the effect of oral NAM in patients with AK. The primary endpoint was the evaluation of the efficacy of intermittent treatment with topical 5-fluorouracil 5% (5-FU 5%) and oral NAM in skin field cancerization. Patients were randomized into four groups according to single or combined therapy with NAM, topical 5-FU, and placebo. As for our study, only patients treated with NAM were considered. Eighteen patients received 500 mg of oral NAM twice daily for 12 months and were assessed for AK counts in a standardized area of the forearms. At the end of the study, the relative reduction in AK count was 13% compared to the baseline [37].

**Table 3:** Descriptive features of other studies on nicotinamide and actinic keratoses.

Study	Phase	n.	Route	NAM, 500 mg	Therapeutic Scheme	Follow-Up (Months)	Relative Reduction of AK with Nicotinamide Compared to Baseline
Surjana et al. (Study 1) (2012) [31]	II	18	Oral	Yes	Twice a day	4	35% (95% CI: 18–48%); *p* = 0.0006
Surjana et al. (Study 2) (2012) [31]	II	21	Oral	Yes	Once a day	4	29% (95% CI: 11–44%) *p* = 0.005
Moloney et al. (2010) [38]	II	13	Topic	No	Twice a day	6	24.6% ± 9.6, *p* = 0.06
Ferreira et al. (2021) [37]	II	18	Oral	Yes	Twice a day	12	Total clearance 23.5%, Partial clearance 83.3%
Chen et al. (ONTRAC) (2015) [32]	III	193	Oral	Yes	Twice a day	12	13% *p* = 0.001
Allen et al. (ONTRANS) (2023) [33]	III	NA	Oral	Yes	Twice a day	12	difference between NAM and placebo group 0.4 (95% CI, −3.0 to 3.7)

AK: actinic keratosis; CI: confidence interval; NA not available; NAM: nicotinamide; ONTRAC: Oral Nicotinamide to Reduce Actinic Cancer; ONTRANS: Oral Nicotinamide to Reduce Actinic Cancer after Transplant; PY: publication year; N.: number of patients included.

To our knowledge, only one study explored the impact of topical NAM on AK. In this randomized, double-blind, placebo-controlled study by Moloney et al., thirteen patients used topical 1% NAM on affected areas of the face, forearms, and scalp twice a day for six months. After six months, the relative reduction of AKs was 22.4% ± 9.6, *p* = 0.06 [38].

In the study ONTRAC [32], the number of AK was 11% lower in the NAM group vs. the placebo group at three months (*p* = 0.01), 14% lower at six months (*p* < 0.001), 20% lower at nine months (*p* < 0.001), and 13% lower at twelve months (*p* = 0.001).

In the ONTRANS trial [33], 153 participants were evaluated for AK, resulting in a 0.4 different rate of AKs between the NAM group vs. placebo group, which was not statistically significant (95% CI, −3.0 to 3.7).

## 4. Dietary Niacin Intake and NMSC

**Figure 4:**
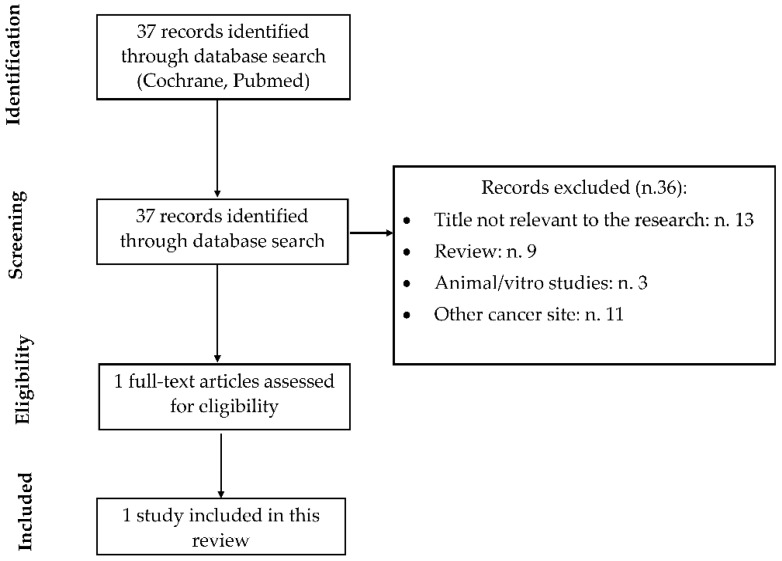
Study flow-chart for the process of selecting the enrolled studies evaluating dietary niacin intake and NMSC.

We systematically searched for relevant published epidemiological studies to assess the association between niacin dietary intake and NMSC. The literature search in the databases returned 37 items (Figure 4). Park et al. [39] supported a potential beneficial role of niacin intake regarding SCC but not BCC or melanoma. The authors prospectively evaluated whether total, dietary, and additional niacin intake was associated with cutaneous cancer risk in the Nurses’ Health Study (1984–2010) and the Health Professionals Follow-up Study (1986–2010). They recorded 23,256 BCC, 2530 SCC, and 887 melanoma cases during the follow-up. Total niacin intake was inversely associated with SCC risk (pooled HR 0.84, 95% CI = 0.74–0.95; *p*-trend = 0.08). However, there was a marginally positive association between total niacin intake and BCC risk (pooled HR 1.05, 95% CI = 1.01–1.10; *p*-trend < 0.01).

## 5. Dysregulation of Nicotinamide N-Methyltransferase and Skin Cancer

Nicotinamide N-methyltransferase (NNMT) exerts a meaningful role in cancer progression. The primary substrate of NNMT is NAM; NNMT irreversibly methylates NAM, generating MNA which cannot enter the NAD+ salvage path, impacting the intracellular NAD+ level. As noted above, while NAM exerts chemopreventive effects, the upregulation of NNMT enormously facilitates cell proliferation, migration, and invasion [40].

Accordingly, NNMT overexpression was reported in numerous neoplasms, including clear cell renal cell carcinoma, oral squamous cell carcinoma, non-small cell lung cancer, and bladder cancer. Moreover, increasing evidence regarding the involvement of this enzyme in skin cancer is emerging: NNMT decreases the intracellular levels of NAM, resulting in UV sensitization of cells and harming the mechanisms implicated in cell cycle arrest and DNA repair. Several studies have explored the role of NNMT in skin cancer: a significantly higher NNMT expression was observed in cutaneous melanoma [41] and BCC specimens [42]. An alteration of NNMT level was also noticed in cutaneous SCC specimens compared to normal tissues. Overexpression of NNMT was also exhibited in AK, even higher than cutaneous SCC [42]; for a review on the role of NNMT in skin cancer, see the stimulating paper by Campagna and coworkers [40].

In light of the considerations mentioned above, the suppression of NNMT may prevent neoplastic transformation and tumor progression, and therefore, NNMT has attained appeal as a promising molecule for targeted therapy [43,44].

## 6. Discussion

Our meta-analysis showed that the NAM consumption appears to exert no effect against SCC, BCC, and NMSC risk: the mean number of BCC and SCC per participant during the intervention period was not statistically different in NAM and in the control arm from the four RCTs published.

The first two published clinical trials [31,32] showed a significant NAM immunoprotective and anti-inflammatory activity against NMSCs, but overall, this effect was not confirmed. The first study by Surjana et al. [31] was conducted on healthy, immunocompetent subjects to determine whether oral NAM, at different doses (500 mg twice or once daily), reduced AK in sun-damaged individuals. The treatment lasted four months, and only two patients in the NAM group developed skin cancers (two cases of BCC and two cases of SCC). The rate ratio of NMSC was 0.24 (*p* = 0.01). The second study was conducted in 2015 by Chen et al. [32], enrolling 193 Australian immunocompetent patients (122 males and 71 females) to receive 500 mg of NAM twice daily for a twelve-month intervention period. Considering continuous covariates, the authors observed a 23% reduction in NMSC incidence [95%CI, 4 to 38]. This result was evident in patients with more NMSCs in the previous five years. There was a lower rate of SCC, estimated at 30% (95% CI, 0 to 51, *p* = 0.05). The effect of NAM was independent of the histological type of SCC (well, moderately, or poorly differentiated). The reduction of BCC incidence was not statistically significant. The follow-up period of six months after the discontinuation of NAM revealed no difference in the rate of NMSC between the two groups.

In 2016, Chen et al. [34] published new data about chemoprevention with oral NAM in immunosuppressed, kidney-transplanted patients. The study was conducted on eleven renal transplant patients receiving 500 mg of NAM twice a day for six months. The recruited patients had a positive personal history of NMSC in the last twelve months. The study did not demonstrate statistically significant results at the end of the treatment period. However, about a 35% relative difference in the rate of NMSCs was achieved compared to the placebo group (*p* = 0.36). In addition, no statistically significant data were obtained considering BCC and SCC in two subgroups.

Recently, another study was conducted on immunosuppressed patients [33]. Allen et al. conducted a Phase III trial to assess the role of oral NAM in the chemoprevention of keratinocyte cancers in solid-organ transplant patients (kidney, liver, heart, or lung). Fifty-nine males and twenty females were enrolled with at least two histologically confirmed keratinocyte cancers. The dose of 500 mg of NAM twice daily for twelve months did not lead to fewer keratinocyte cancers or AK in these patients. The rate ratio of keratinocyte cancer was 1 (*p* = 0.96), and the incidence of SCC and BCC was similar in the two studied groups.

Only two out of four studies analyzing the protective role of NAM showed a significant effect on the rate of new keratinocyte cancers [31,32]. The results of the other two studies, which failed to demonstrate a benefit in the NAM patients, could be explained by different factors, i.e., the paucity of tumor lesions detected (which were below the expected number of tumors as suggested by previous studies), and the short intervention period: six months in the phase II trial [31] and twelve months in the ONTRANS trial, respectively [31]. The limits of these two trials are probably related to the condition of immunosuppression of enrolled patients and the slow recruitment. In addition, in the ONTRANS trial, previous use of NAM supplementation four weeks prior to study entry was not an exclusion criterion [32]. Considering the Australian practice of clinicians prescribing oral NAM to immunosuppressed patients, some enrolled transplanted patients with a high risk for keratinocyte cancers already used NAM, thus reducing their real risk for NMSC [33].

The paper by Chen et al. [34], with a six-month follow-up, showed an increase in new NMSCs after the therapy discontinuation, thus emphasizing that the chemopreventive activity of NAM does not exert a long-term effect and ends with its interruption.

In the trials included in the present meta-analysis, adherence to the treatment among patients was excellent (78–98%). On the contrary, in three of them, only half of the patients used sunscreen, showing that the topical prophylactic therapy appears unsatisfactory, mainly due to poor adherence to the sunscreen application. On the other hand, patients preferred oral chemoprophylaxis due to easier intake, self-administration, limited therapy duration, and affordability.

Oral consumption of NAM showed excellent tolerability with few side effects up to a dose of 3 mg daily [45]. Unlike niacin, oral NAM does not have vasodilatory effects and is not associated with cutaneous flushing [46]. As a chemopreventive treatment, 1 g daily of NAM does not show any significant adverse effects or changes in weight, blood pressure, total blood count, creatinine, liver, or kidney function compared to placebo. In the literature, only a few cases of reversible hepatotoxicity with ten g/day or nine g/day have been reported, wholly resolved at the interruption of therapy [46].

In the literature, the immunoprotective activity of NAM has been explored in two controlled trials [47,48] on 91 healthy patients who received 500 mg NAM twice or thrice daily. Overall, these studies showed increased skin immunity by using the Mantoux model. Patients underwent photodynamic therapy, and a Mantoux test was performed in the irradiated areas. The NAM group presented an excellent response to the Mantoux test compared to the placebo group, with a statistically significant reduction of UV-induced immunosuppression.

Physiological levels of NAM are partly a result of daily dietary intake, thanks to its precursor niacin, found in a broad spectrum of foods. According to the Scientific Opinion on Dietary Reference Values for niacin published by the EFSA Panel [49], the recommended intake was set at 1.6 mg NE/MJ. This would correspond to an intake of about 13–15 mg NE/day for women and 15–19 mg NE/day for men [49]. Its deficiency might cause pellagra, a disease characterized by the four Ds, diarrhea, dermatitis, dementia, and death. Drugs, alcoholism, gastrointestinal tract diseases, malignancies, and medications such as isoniazid are causes of secondary pellagra [50].

The EFSA [49] estimated the dietary intake of niacin using their comprehensive Food Consumption Databases [51]. At the European level, the average total niacin intakes in adults ranged from 27–53 mg NE/day, and ranges varied from 11.7–31.3 mg NE/day at the lower end (5th percentile) to 37.3–78.2 mg NE/day at the upper end (95th percentile). Average daily intakes (but not energy-adjusted) were slightly higher among males than females, mainly due to more significant amounts of food consumed.

The main food groups contributing to niacin intakes included meat and meat derivatives, grains and grain-based products, and milk and dairy products. Other essential food groups contributing to niacin intake in adult men were composite dishes, fish and fish products, coffee and cocoa beverages, starchy roots or tubers, and alcoholic beverages [49]. In addition, humans can meet their niacin requirement by ingesting nicotinic acid, NAM, nicotinamide riboside, and tryptophan. Plant products contain mainly nicotinic acid, while animal products provide NAM from its nucleotide forms during food processing. Dietary tryptophan can be a substrate to create endogenous niacin in a 60 mg tryptophan to 1 mg niacin ratio [52].

Recent studies found that dietary niacin intake may be significant in cancer risk [53]. We found only one paper investigating the association between dietary intake of niacin and NMSC risk [39]. The authors found a potentially beneficial role of niacin intake concerning the risk of SCC, but not in BCC or melanoma, in a sample of the US population. The hypothesis is that niacin might protect the skin against UV radiation-induced DNA damage through diverse pathways, including DNA repair, genomic stability, and transcription [48,54]. However, the biological pathways could be different compared to BCC. In the US population, multivitamin supplements are widespread, and the total niacin intake was close to the Recommended Daily Allowance (RDA) of niacin (16 mg/day for adult males and 14 mg/day for adult females). The authors did not exclude the possibility that other components in multivitamins might have masked BCC’s inverse association. Moreover, they commented that over 80% of their participants consumed over the RDA of niacin. However, the mean values of the top quintiles of total niacin intake were far lower than the dose typically used in the clinical trials.

Interestingly, Ying and coworkers [55], using NHANES data from 1999 to 2014, investigated the association between dietary niacin intake and mortality in cancer patients. They found that niacin intake was negatively associated with mortality outcomes in patients with cancer, and the association persisted in subgroup analyses based on sex, age, and body mass index. The Kaplan–Meier curves showed that groups with higher niacin intake had better survival rates than low-intake groups. Niacin supplementation improved cancer mortality but not all-cause mortality. Niacin may reduce DNA damage and carcinogenesis, affecting metastases, recurrences, and survival because NAD is consumed as a substrate in the adenosine diphosphoribose transfer reaction in response to carcinogen-induced DNA damage [22].

As for other types of cancers than NMSC, there are contrasting results. No association was observed between dietary niacin intake and cervical intraepithelial neoplasia [56], colorectal [57,58], and esophageal cancer risk [59]. The results could be more consistent with the assessment of niacin deficiency, niacin intake, or confounding factors, such as genetic polymorphism, or by the effect of other dietary, lifestyle, or undefined factors [49].

In other studies, dietary niacin intake was inversely associated with squamous cell cancer of the esophagus [60,61] and hepatocellular carcinoma [62]. In other studies, dietary niacin intake was positively associated with cervical intraepithelial neoplasia [63], pancreas [64], and colorectal cancer [65].

However, the exact mechanisms remain to be understood. Niacin might influence DNA repair, genomic stability, and the immune system, eventually impacting cancer risk, but its metabolism requires complex interactions [52]. Moreover, it can be influenced by numerous factors such as income, education level, race, and comorbidity [66]. To date, identifying the correct dose for cancer prevention is challenging since niacin intake is highly variable across the population, exceeding the tolerable upper intake level [66], and some uncertainty exists about the potential benefit, even in other conditions (diabetes, cardiovascular disease, and neurological disorders) [52,66,67,68]. Further research is needed.

## 7. Conclusions

Our meta-analysis did not reveal a significant positive effect of oral NAM as a chemopreventive agent for NMSCs. This result could be partially explained by the fact that two out of four clinical trials in our metanalysis included immunocompromised patients. It is well known that immunocompromised patients have a higher risk of NMSC, so this could explain why NAM did not work in these subsets of patients. Moreover, the early discontinuation of the recruitment of patients in the ONTRANS trial could further influence the results of oral NAM in this cohort of patients. As also highlighted by Schmults et al. [69], in the ONTRANS trial, there was a 24% reduction in the occurrence of dermal invasive cutaneous SCC, which is not negligible.

However, considering the studies of Surjana 2012 [31] and Chen 2015 [29], where immunocompetent patients were enrolled, we noticed that oral NAM produced an impact, i.e., a reduction in the number of NMSCs in the two studies. The protective effect of NAM was more evident in the trial by Surjana et al. [31] and less prominent in the study by Chen et al. [32].

In conclusion, additional studies on larger populations and over a more extended period are required to establish the real potential of NAM in the chemoprevention of NMSC and investigate the dietary intake of niacin and supplements.

Considering the favorable safety profile and excellent adherence to treatment observed in clinical trials and real-world experiences, and the promising results of oral NAM in the studies on immunocompetent patients, chemoprevention with oral NAM might be considered in subjects with a positive history or at higher risk of NMSC.

## Figures and Tables

**Table 1:** Main characteristics of the studies included in the review that reported the association between the use of NAM and the incidence of new NMSC in high-risk patients.

Study	Study Name and Country	Phase	NAM: Placebo n.	Mean Age	Sex M:F	Immunos.	TD ¥	Compliance NAM	Rate Ratio NMSC (95% CI)	Rate Ratio BCC (95% CI)	Rate Ratio SCC (95% CI)	BCC, n.	SCC, n.
Surjana et al. (2012) ** [31]	-, AUS	II	37:37:00	67–72	15:3; 14:7	no	4	94–98%	0.24			14	10
									(0.08–0.71)				
**Chen et al. (2015)** [32]	ONTRAC, AUS	III	193:193	66.4	122:71	no	12	88%	0.77	0.8	0.7	239	97
									(0.62–0.95)	(0.61–1.06)	(0.49–1.00)		
**Chen et al. (2016)** [34] *	-, AUS	II	11:11	65	09:02	yes	6	93–98%	0.65	0.33	1	7	23
									(0.30–1.60)	(0.10–1.5)	(0.50–2.5)		
**Allen et al. (2023)** [33]	ONTRANS, AUS	III	79:79	62.2	59:20:00	yes	12	78%	1	1.4	0.9	69	138
									(0.80–1.30)	(0.80–2.30)	(0.60–1.20)		

TD: treatment duration; M: male; F: female; Immunos.: immunosuppressed participants; ONTRAC: Oral Nicotinamide to Reduce Actinic Cancer; ONTRANS: Oral Nicotinamide to Reduce Actinic Cancer after Transplant; NAM: nicotinamide; NMSC: non-melanoma skin cancer; BCC: basal cell carcinoma; SCC: squamous cell carcinoma. * NAM was given in two different dosages (1000 daily, 500 daily); ** data obtained from personal communications. ¥ in months.

**Table 2:** Risk of bias and GRADE.

Study	Random Sequence Generation	Allocation Concealment	Blinding of Participants and Personnel	Blinding of Outcome Assessment	Incomplete Outcome Data	Selective Reporting	Other Bias	GRADE
Surjana et al. (2012) [31]	L	L	L	L	L	L	L	M
Chen et al. (2015) [32]	L	L	L	L	L	L	L	H
Chen et al. (2016) [34]	L	L	L	L	L	L	L	M
Allen et al. (2023) [33]	L	L	L	L	L	L	L	H

L: low risk, H: high risk,. GRADE. M: moderate, phase II trials; H: high: phase III trials.

## Data Availability

The datasets generated during and/or analyzed during the current study are available from the corresponding author on reasonable request.
